# Association analysis of *PAEP*, *KRT*10, and *BMP*7 genes SNPs with reproductive traits in Kele pigs

**DOI:** 10.1371/journal.pone.0311064

**Published:** 2025-07-14

**Authors:** Yong Zhao, Chunyuan Wang, Yan Wu, Jin Xiang, Yiyu Zhang

**Affiliations:** 1 Key Laboratory of Animal Genetics, Breeding and Reproduction in the Plateau Mountainous Region, Ministry of Education, College of Animal Science, Guizhou University, West Campus, Guiyang, Guizhou, People’s Republic of China; 2 Institute of Xiang Pigs, Guizhou University, West Campus, Guiyang, Guizhou, People’s Republic of China; International Medical University, MALAYSIA

## Abstract

This study aimed to investigate the effects of single nucleotide polymorphisms (SNPs) in progestogen-associated endometrial protein (*PAEP*), keratin 10 (*KRT*10), and bone morphogenetic protein 7 (*BMP*7) genes on reproductive traits (total number of piglets born, number of piglets born alive, litter birth weight, number of piglets weaned and litter weight weaned) in Kele pigs. We used 255 multiparous Kele sow (2–4 parities) as research materials. SNPs were identified by using a PCR amplification instrument and sequence alignment software DANMAN. The population genetic characteristics of SNPs were analyzed using the SHEsis online software. Bioinformatics analyses of SNPs were conducted using RNAfold, SOPMA, SWISS-MODEL, and Swiss-PdbViewer programs. The associations between the SNPs and reproductive traits in Kele pigs were analyzed through SPSS 22.0 software. In this study, nine SNPs were identified in the three genes: g.1884992 T > C (exon 4), g.1885125 G > C (intron 4), and g.1885158 G > A (intron 4) in *PAEP*, g.21643703 C > T (intron 4), g.21643714 G > A (intron 4) and g.21643741 G > A (exon 5) in *KRT*10, and g.57647887 G > A (intron 3), g.57647990 C > T (intron 3) and g.57648145 C > G (intron 3) in *BMP*7. In SNPs g.1884992 T > C of *PAEP,* missense mutation eventuated structural changes in mRNA and proteins’ secondary structure. In SNPs g.21643741 G > A of *KRT*10*,* the synonymous mutation led to an alteration in mRNA secondary structure. For *PAEP,* the CC genotype in SNPs g.1885125 G > C and the AA genotype in SNPs g.1885158 G > A showed significantly higher values than other genotypes in all reproduction traits except for litter birth weight, preliminarily identified as the favorable genotypes. For *KRT*10, the GG genotype in SNPs g.21648641 G > A showed significant superiority to the AA genotype (*P < *0.05) in all aspects except for litter birth weight and notably surpassed the GA genotype in the total number of piglets born (*P < *0.05), preliminarily recognized as a favorable genotype. Regarding *BMP*7, the GA genotype in SNPs g.57647887 G > A and the CT genotype in SNPs g.57647990 C > T exhibited significantly higher number of piglets born alive and number of piglets born alive compared to other genotypes (*P < *0.05). The GG genotype in SNPs g.57648145 C > G was significantly associated with higher litter birth weight (*P < *0.05). The result of diplotype analyses indicated that the H3H3 (CCGGGG) of *PAEP* and H3H3 (CCGGAA) of *KRT*10 had a significant effect on the five traits. For *BMP*7, the H4H4 (AATTGG) diplotype showed a significant influence on all genotypes except litter birth weight.

## Introduction

Kele pigs, native to the Yunnan-Guizhou Plateau, have excellent meat quality with strong water retention, juicy muscle, and good color and are an excellent ingredient for Xuanwei ham. However, they are accompanied by poor reproductive traits, such as low litter size and teat number [1,2]. Reproductive efficiency has a great impact on the economic benefit of pork production [3]. With the improvement of total living standards, there is an increasing global demand for pork [4], which precipitates the pig farming industry has become more and more prosperous. The poor reproductive traits of Kele pigs have made it hard to meet the demands of pork yields in modern society [5], which severely impacts their development. Therefore, improving the reproductive traits of Kele pigs holds practical significance for enhancing efficiency and sustainability, increasing production efficiency, and meeting market demands.

*PAEP*, also known as the β-lactoglobulin (*BLG*) gene, spans 4193 bp, including 7 exons and 6 introns [6]. The protein encoded by this gene consists of 180 amino acids and belongs to the Nuclear Lipid Calcium-Binding Proteins Family [7]. As a secretory protein usually expressed in the reproductive tract with anti-tumor growth characteristics, PAEP has been primarily studied in reproductive system diseases [8–10]. In addition, β-lactoglobulin coding genes often have a significant effect on milk traits [11,12]. A large number of authors have reported the study of *BLG* polymorphism on milk production traits and milk quality traits [13,14]. Although no studies have been reported in pigs, previous research found that *PAEP* polymorphisms significantly affected reproductive traits in cattle, such as calving interval and gestation length [15,16]. The impact of *PAEP* polymorphisms on reproductive traits has been observed in livestock species, suggesting its potential functional role in fertility. Therefore, the impact of *PAEP* gene polymorphisms on reproductive traits in pigs warrants further investigation.

*KRT*10, located on chromosome 12 of a pig, with 8 exons and 7 introns, is a gene encoding keratin 10 (K10). Keratin 10 is a type I keratin, as the main structural protein of the epidermis [17]. Nowadays, the research on *KRT*10 has been focused on various disease mechanisms [18,19], especially the regulation of ichthyosis [20–23]. Furthermore, Wu et al. [24] found that phosphatase and tensin homolog (PTEN) overexpression improved the cisplatin-resistance of human ovarian cancer cells through upregulating *KRT*10 expression. In the study by Gao et al. [25], the downregulation of *KRT*10 gene expression may be involved in the inflammatory response during the development of mastitis in sheep. Cunha et al. [26] found *KRT*10 expression is induced following the conversion of the vaginal epithelium into glycogen-rich squamous epithelium by endogenous estrogen, suggesting its involvement in epithelial differentiation and hormonal regulation. These findings suggest that genetic variations in this gene could potentially affect epithelial integrity, immune homeostasis, and tissue remodeling in the reproductive tract. Given the critical role of these processes in reproductive function, future studies should explore the functional consequences of *KRT*10 variation and their potential impact on reproductive efficiency.

*BMP*7, also known as bone morphogenetic protein-7, is a member of the transforming growth factor beta (TGF-β) supergene family of functional proteins [27], and it was identified by Celeste et al. [28]. *BMP*7 can induce undifferentiated mesenchymal cells in the perivascular and connective tissues to differentiate into bone and cartilage cells, leading to the formation of bone tissue, playing a crucial role in the regulation of bone homeostasis and vertebral development [29,30]. *BMP*7 is expressed in the ovary, uterus, and mammary glands of livestock and poultry [31–35]. Scientists have suggested that *BMP*7 may be essential for normal folliculogenesis and ovulation [32]. Furthermore, the decrease in *BMP*7 expression may facilitate decidulization of the endometrium, thus aiding the establishment of pregnancy [33]. In the study of Ying et al. [34], they suggested that *BMP*7 may antagonize transforming growth factor β1 (TGFβ1) in tumorigenesis-associated epithelial-mesenchymal transition (EMT) in breast cancer, so *BMP*7 may be a promising therapeutic target for treating breast cancer. *BMP*7 also exhibits high expression levels in cervical cancer, making it a potential therapeutic target for cervical cancer treatment [35]. Therefore, considering these functional roles, *BMP*7 may influence reproductive traits through molecular pathways involved in ovarian follicular development, endometrial remodeling, and cellular differentiation. Furthermore, Li et al. [36] found that *BMP*7 was identified to contribute to sow reproductive traits by whole-genome sequencing and merit further investigations. In the study of Yin et al. [37], *BMP*7 was confirmed as a candidate gene for porcine reproductive traits.

Based on the regulatory significance of *PAEP*, *KRT*10, and *BMP*7 in reproductive physiology, we tried to analyze the associations between SNPs and reproductive traits in Kele pigs, aiming to provide genetic markers for the reproductive traits of Kele pigs and expedite breeding progress.

## Materials and methods

### Experimental materials

The experimental pig population was selected from the Kele pig farm in Hezhang County, Guizhou Province, China. They were a group of multiparous sows (2–4 parities) in the same feeding environment, and they were healthy and disease-free. A total of 255 multiparous sows of the same age (450 days) were randomly chosen, and approximately 0.5 g of ear tissue was collected from each sow for extracting DNA. We locally injected an anesthetic Zoletil® 50 (Batch No. 7LR9A, Virbac, France) into the ear region of the pigs, following the guidelines provided in the manufacturer’s instructions. Once the anesthetic took effect, ear tissue was swiftly and precisely collected to minimize discomfort. Postoperatively, the pigs were administered analgesics to alleviate pain, and appropriate postoperative care was provided to prevent infection. This experiment was approved by the animal ethics committee of Guizhou University (EAE-GZU-2022-P049). The collected ear tissue samples were temporarily preserved in cryovials containing 75% ethanol, with the corresponding sow ear tag numbers recorded sequentially on each tube, and then promptly transported to the laboratory and stored at −40°C for subsequent use. The first-parity reproductive data of 255 pigs were measured and recorded at the pig farm: total number of piglets born, number of piglets born alive, litter birth weight, number of piglets weaned, and litter weight weaned of the first birth. The weaning age of these experimental Kele pigs was 28 days.

### DNA extraction

The ear tissue was retrieved from the freezer, washed thoroughly with sterile phosphate-buffered saline (PBS) to remove residual contaminants, and finely chopped using sterilized scissors. Approximately 20 mg of tissue was transferred to a 1.5 mL microcentrifuge tube for DNA extraction. The DNA was extracted using the Genomic DNA Extraction Kit (Dalian Bao Biotechnology Co., Ltd, Guiyang, China), following the manufacturer’s protocol. The quality of the extracted DNA was assessed using 1.5% agarose gel electrophoresis to check for integrity and potential degradation, with qualified samples displaying a single, high-molecular-weight amplicon without smearing or degradation. A Nanodrop 2000c spectrophotometer (Thermo Fisher Scientific Co., Ltd, Shanghai, China) was used to measure DNA purity based on the A260/A280 ratio. The ratio of qualified DNA samples was typically between 1.7 and 1.9. Then, the qualified DNA samples were diluted to 150ng/ μL and stored at −20°C for later use.

### Primer design

The DNA reference sequence of the *PAEP*, *KRT*10, and *BMP*7 genes were obtained from the NCBI database (accession number: NW_018084833.1, NC_010454.4, NC_010459.5). Primers were designed using the online software Premier 5.0 (Table 1). To ensure specificity and efficiency in PCR amplification, we considered several critical parameters. Primer length was 18−22 base pairs. The optimal Tm for primers is between 52°C and 62°C. GC content is 40−60%. All designed primer sequences were synthesized by Shenggong Biotechnology Co., Ltd (Shanghai, China) and then stored at −20°C for later use.

**Table 1:** Primer sequence information of *PAEP*, *KRT*10, *BMP*7.

Primer		Primer sequences (5'→3')	Primer sequences/Size (bp)	Temperature (°C)
*PAEP*	F1	GATTGATTGGCGAATGTGC	g.1884633~1885248	60
	R1	CTCGGATACAAAAACCTCTTT			616bp
*KRT10*	F1	TGTATGCCACCATAAAAGCC	g.21643150~21643743	56	
	R1	CAGTGTGACTACAGTGAGCC			594bp
	F2	CTACCTGAAGAAGAACCACGA	g.21643720~21644244	58	
	R2	TCAGCACTTTTAGATGAATGCC			525bp
*BMP7*	F1	AGGCTTCGTAAACCCCAATC	g.57647662~g.57648174	58	
	R1	TGGGAACCCCTAATTCACTC			513bp

### PCR amplification and SNP detection

PCR was performed in a total of 20 volumes of μL, including the following: 1 μL DNA, 10 μL 2 × Taq PCR Master Mix, 1 μL forward and reverse primers each (10 μmol/L), and 7 μL ddH_2_O. The PCR reaction procedure included initial denaturation at 94°C for 8 min, followed by 32 cycles of denaturation at 94°C for 30 s, annealing at the optimal temperature for 30 s, then extension at 72°C for 30 s, and final extension at 72°C for 8 minutes. The PCR products were analyzed via 1.5% agarose gel electrophoresis. PCR products with clear, bright, and free of redundant amplicons were sent to Shenggong Biotechnology Co., Ltd (Shanghai, China) for sequencing. Sequencing results were used for SNP identification and genotyping through Chromas [38], Editseq software [39], and our manual calculations.

### Statistical analyses

Allelic frequency, genotype frequency, heterozygosity (*He*), polymorphism information content (*PIC*), number of effective alleles (*Ne*), and Hardy-Weinberg equilibrium were calculated by EXCEL2010 software [38]. SHEsis online software (http://analysis.bio-x.cn/) was applied to calculate and analyze linkage disequilibrium (LD) and haplotypes at SNPs [39]. The multifactorial variable analysis of the general linear model (GLM) was used for the correlations between SNPs and reproductive traits, as well as the association between diplotypes and reproductive traits. Multiple comparisons were conducted using Duncan’s method [39,40]. Data results were presented as mean ± standard deviation. All statistical analyses were performed using SPSS 23.0 software with the independent (unpaired) samples t-test [38]. Mutations’ effects on mRNA secondary structure, protein secondary structure, protein tertiary structure, and local structure were analyzed using online tools, including RNAfold (http://rna.tbi.univie.ac.at/cgi-bin/RNA WebSuite/RNA fold. cgi), SOPMA (https://npsa-prabi.ibcp.fr/cgi-bin/npsa_automat.pl?page=npsa%20_sopma.html), and SWISS-MODEL (https://swissmodel.expasy.org/interactive), as well as the Swiss-PdbViewer software [38,39].

## Results

### Summary of mutation sites

**Fig 1:**
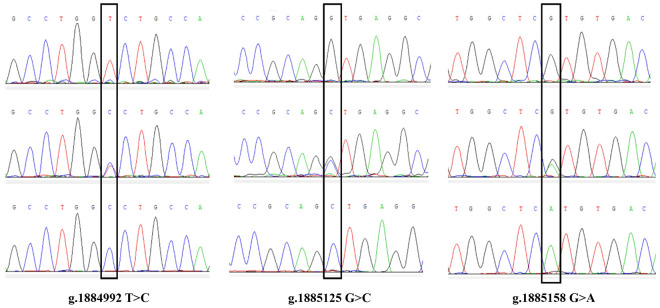
The sequence peak map of the SNPs in the *PAEP* gene.

**Fig 2:**
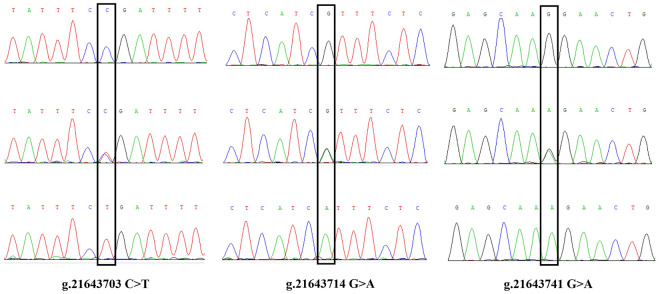
The sequence peak map of the SNPs in the *KRT10* gene.

**Fig 3:**
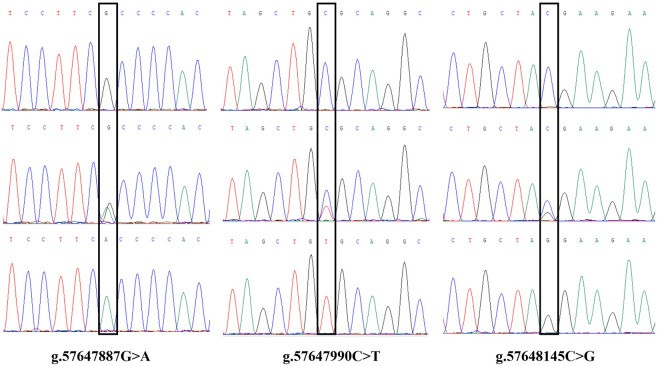
The sequence peak map of the SNPs in the *BMP7* gene.

We identified 9 SNPs totally and obtained the following peak maps according to the sequencing results (S1-S3 Appendixes), and the maps were displayed in Figs 1–3. The SNPs g.1884992 T > C located in the fourth exon, g.1885125 G > C and g.1885158 G > A were detected in the fourth intron of *PAEP*. The SNPs g.21643703 C > T and g.21643714 G > C were located in the fourth intron, and g.21643741 G > A was located in the fifth exon of *KRT*10. The SNPs g.57647887 G > A, g.57647990 C > T, and g.57648145 C > G were all located in the third intron of *BMP*7. The g.1884992 T > C missense mutation of *PAEP* led a codon to change from GTC to GCC, causing the amino acid encoded at position 135 to transition from Valine (V) to Alanine (A). The g.21643741 G > A mutation in *KRT*10 changed the codon from AAG to AAA, encoding Lysine (K) in both cases, representing a synonymous mutation.

### Population genetic information analysis

We counted the gene frequencies, genotype frequencies, and genetic indexes of the 9 SNPs, and the results were shown in Table 2. The dominant genotypes and frequencies of the SNPs g.1884992 T > C, g.1885125 G > C, and g.1885158 G > A were TT (0.68), GG (0.60), and GG (0.60) respectively, and the dominant alleles and frequencies were T (0.65), G (0.73), and G (0.73). The result of genetic index analysis revealed that the number of effective alleles of the SNPs g.1884992 T > C, g.1885125 G > C, and g.1885158 G > A were 1.46, 1.66, and 1.66, suggesting moderate genetic diversity, with SNP g.1884992 T > C showing slightly higher variation compared to the other two SNPs. The heterozygosity values of SNPs g.1884992 T > C, g.1885125 G > C, and g.1885158 G > A were 0.69, 0.40, and 0.40, respectively. This indicated a high level of heterozygosity at the SNP g.1884992 T > C, with a large proportion of individuals carrying different alleles, while SNPs g.1885125 G > C and g.1885158 G > A exhibited moderate heterozygosity. Polymorphism information content analysis revealed that the PIC values of SNPs g.1884992 T > C, g.1885125 G > C, and g.1885158 G > A were 0.26, 0.32, and 0.32, representing SNPS g.1885125 G > C and g.1885158 G > A belong to intermediate polymorphism (0.25 < *PIC* < 0.5). The SNP g.1884992 T > C had low polymorphism (*PIC < *0.25), indicating a limited genetic variability. Chi-square tests indicated that SNPs g.1884992 T > C, g.1885125 G > C, and g.1885158 G > A highly significantly deviated from Hardy-Weinberg equilibrium (*P < *0.01), which meant the observed genotype frequencies differ statistically from the expected frequencies based on allele distribution.

**Table 2:** Population genetic information of *PAEP, KRT*10, and *BMP*7 SNPs in Kele pigs.

SNPs	Genotype frequency			Allele frequency		*Ne*	*PIC*	*χ* ^2^	*P*	*He*
**g.1884992 T > C**	TT (173)	TC (65)	CC (17)	T	C	1.46	0.26	8.75	0.003	0.69
	0.68	0.25	0.07	0.65	0.35					
**g.1885125 G > C** **/1885158 G > A**	GG/GG (153)	GC/GA(65)	CC/AA (37)	G/G	C/A	1.66	0.32	32.53	0.001	0.40
	0.60	0.25	0.15	0.73	0.27					
**g.21643703 C > T**	CC (115)	CT (113)	TT (27)	T	C	1.79	0.34	0.01	0.920	0.44
	0.45	0.44	0.11	0.67	0.33					
**g.21643714 G > A**	GG (200)	GA (45)	AA (10)	G	A	1.29	0.20	10.88	0.001	0.22
	0.78	0.18	0.04	0.87	0.13					
**g.21643741 G > A**	GG (162)	GA (78)	AA (15)	G	A	1.50	0.28	1.79	0.181	0.33
	0.65	0.31	0.06	0.79	0.21					
**g.57647887 G > A/** **g.57647990 C > T**	GG/CC (95)	GA/CT (102)	AA/TT (58)	G/C	A/T	1.96	0.37	8.52	0.004	0.49
	0.39	0.44	0.17	0.57	0.43					
**g.57648145 C > G**	CC (79)	CG (106)	GG (70)	C	G	2.00	0.38	7.16	0.008	0.50
	0.31	0.42	0.27	0.52	0.48					

Note: He is observed heterozygosity, Ne is the effective number of the alleles, PIC is the polymorphic information content, χ^2^-HWE is the genotype Hardy-Weinberg equilibrium, χ^2^-0.05 = 5.991, χ^2^-0.01 = 9.210. The next tables are similar.

In the SNPs g.21643703 C > T, g.21643714 G > A, and g.21643741 G > A, the dominant genotypes and frequencies were CC (0.45), GG (0.78), and GG (0.65), and the dominant alleles and frequencies were C (0.67), G (0.87), and G (0.79), respectively. The number of effective alleles of the SNPs was 1.79, 1.29, and 1.50, with heterozygosity values of 0.44, 0.22, and 0.33 respectively, indicating moderate genetic diversity and heterozygosity in SNPs g.21643703 C > T and g.21643741 G > A, and low diversity and heterozygosity in SNP g.21643714 G > A. The SNPs g.21643703 C > T and g.21643741 G > A exhibited intermediate polymorphism (0.25 < *PIC* < 0.5), and g.21643714 G > A showed low polymorphism (*PIC < *0.25). The SNPs g.21643703 C > T and g.21643741 G > A were in agreement with Hardy-Weinberg equilibrium (*P > *0.05), which meant that for the two SNPs in the population, the frequencies of genotypes and alleles remained constant across generations. The SNP g.21643714 G > A highly significantly deviated from Hardy-Weinberg equilibrium (*P < *0.01).

Results showed that for the SNPs g.57647887 G > A, g.57647990 C > T, and g.57648145 C > G, the dominant genotypes and alleles respectively were GA (0.44), CT (0.44), CG (0.42), G (0.57), C (0.57), and C (0.52). The number of effective alleles were 1.96, 1.96, and 2.00, with heterozygosity values of 0.49, 0.49, and 0.50, indicating intermediate heterozygosity. All SNPs exhibited intermediate polymorphism (0.25 < *PIC* < 0.5) and highly significantly deviated from Hardy-Weinberg equilibrium (*P* < 0.01).

### Haplotype and genotype analyses, linkage disequilibrium analysis of *PAEP*

As shown in Table 3, there were 3 haplotypes and 6 diplotypes in *PAEP.* The dominant haplotype was H1 (TGG, 0.53). The dominant diplotype was H1H1 (TTGGGG, 0.37). For the SNPs g.1884992 T > C and g.1885125G > C (Table 4), the D ‘values were 0.618, and r^2^ was 0.023. The D’ value and r^2^ between g.1884992 T > C and g.1885158 G > A were respectively 0.618, 0.023. The D ‘value and r^2^ between g.1885125 G > C and g.1885158 G > A were both 1.000, indicating a complete linkage (D ‘ > 0.85, r^2 ^> 0.33). There was no strong linkage disequilibrium effect between other SNPs.

**Table 3:** Analyses of haplotype and diplotype of *PAEP* gene in Kele pigs.

Type	Genotype	g.1884992T > C	g.1885125 G > C	g.1885158 G > A	Frequency
**Haplotype**	H1 (272)	T	G	G	0.53
	H2 (139)	T	C	A	0.27
	H3 (99)	C	G	G	0.20
**Diplotype**	H1H1 (94)	TT	GG	GG	0.37
	H1H2 (42)	TT	GC	GA	0.16
	H1H3 (42)	TC	GG	GG	0.16
	H2H2 (37)	TT	CC	AA	0.15
	H2H3 (23)	TC	GC	GA	0.09
	H3H3 (17)	CC	GG	GG	0.07

**Table 4:** Linkage disequilibrium analysis of *PAEP* gene in Kele pigs.

SNPs	g.1884992 T > C	g.1885125G > C	g.1885158 G > A
**g.1884992 T > C**	–	0.62	0.62
**g.1885125 G > C**	0.02	–	1.00
**g.1885158 G > A**	0.02	1.00	–

Note: The upper triangle is the D′ values, and the lower triangle is the r^2^ values. The next tables are the same.

### Haplotype and genotype analyses, linkage disequilibrium analysis of *KRT*10

It was observed that four haplotypes and ten diplotypes were in *KRT*10 (Table 5). The major haplotype was H1 (CGG), with the highest frequency of 0.33. The dominant diplotype was H1H2 (CTGGGG), with a frequency of 0.24. The r^2^ values among these SNPs were all lower than 0.33, therefore, there was no strong linkage disequilibrium among the three SNPs (Table 6).

**Table 5:** Analyses of haplotype and diplotype in *KRT*10 gene.

Type	Genotype	g.21643703 C > T	g.21643714 G > A	g.21643741 G > A	Frequency
**Haplotype**	H1 (170)	C	G	G	0.33
	H2 (167)	T	G	G	0.33
	H3 (108)	C	G	A	0.21
	H4 (65)	C	A	G	0.13
**Diplotype**	H1H1 (29)	CC	GG	GG	0.11
	H1H2 (60)	CT	GG	GG	0.24
	H1H3 (35)	CC	GG	GA	0.14
	H1H4 (17)	CC	GA	GG	0.07
	H2H2 (27)	TT	GG	GG	0.11
	H2H3 (34)	CT	GG	GA	0.13
	H2H4 (19)	CT	GA	GG	0.08
	H3H3 (15)	CC	GG	AA	0.06
	H3H4 (9)	CC	GA	GA	0.04
	H4H4 (10)	CC	AA	GG	0.04

**Table 6:** Linkage disequilibrium analysis of *KRT*10 gene in Kele pigs.

SNPs	g.1884992 T > C	g.1885125G > C	g.1885158 G > A
**g.21643703 C > T**	–	0.99	1.00
**g.21643714 G > A**	0.07	–	0.99
**g.21643741 G > A**	0.13	0.04	

### Haplotype and genotype analyses, linkage disequilibrium analysis of *BMP*7

Table 7 showed that four haplotypes and nine diplotypes were in *BMP*7, H1 (GCC) and H1H2 (GACTGC) were the most frequent of them, with the highest rates of 0.37 and 0.22, respectively. The linkage disequilibrium analyses showed the SNPs g.57647887 G > A and g.57647990 C > T had a complete linkage effect, meanwhile, there was no strong linkage disequilibrium effect between the remaining SNPs (Table 8).

**Table 7:** Analyses of haplotype and diplotype in *BMP*7 gene in Kele pigs.

Type	Genotype	g.57647887G > A	g.57647990 C > T	g.57648145 C > G	Frequency
**Haplotype**	H1(187)	G	C	G	0.37
	H2(161)	A	T	C	0.32
	H3(105)	G	C	C	0.21
	H4(57)	A	T	G	0.10
**Diplotype**	H1H1(39)	GG	CC	GG	0.15
	H1H2(57)	GA	CT	GC	0.22
	H1H3(36)	GG	CC	GC	0.14
	H1H4(16)	GA	CT	GG	0.06
	H2H2(31)	AA	TT	CC	0.12
	H2H3(29)	GA	CT	CC	0.11
	H2H4(13)	AA	TT	GC	0.05
	H3H3(20)	GG	CC	CC	0.08
	H4H4(14)	AA	TT	GG	0.06

**Table 8:** Linkage disequilibrium analysis of *BMP*7 gene in Kele pigs.

SNPs	g.57647887 G > A	g.57647990 C > T	g.57648145 C > G
**g.57647887 G > A**	–	1.00	0.64
**g.57647990 C > T**	0.97		0.64
**g.57648145 C > G**	0.23	0.22	–

### The mRNA secondary structure predictions for *PAEP* and *KRT*10 Genes

**Fig 4:**
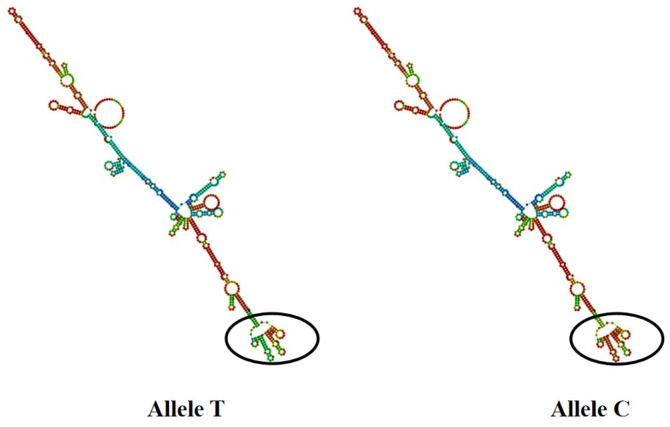
The change of mRNA secondary structure in SNPs g.1884992 T>C for *PAEP.*

**Fig 5:**
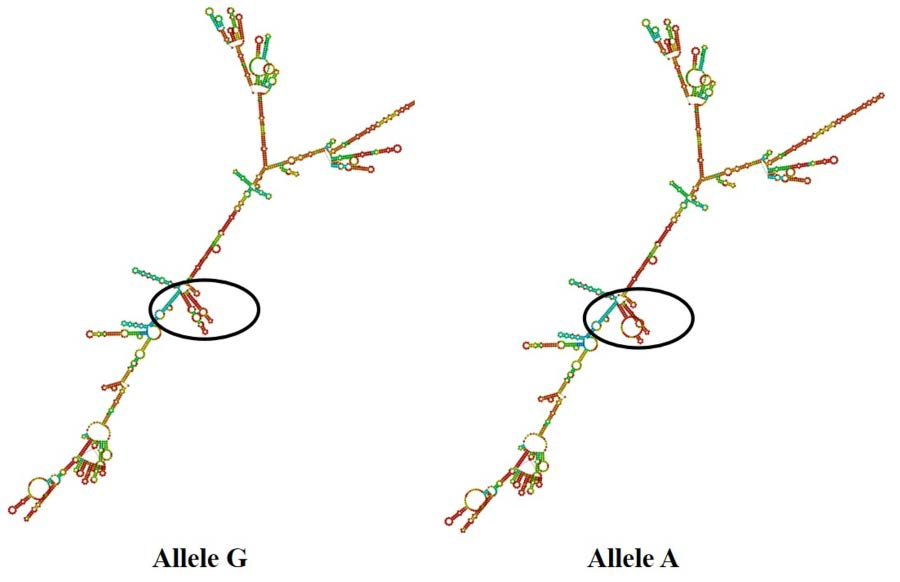
The change of mRNA secondary structure in SNPs g.21643741 G>A for *KRT10.*

We conducted mRNA secondary structure predictions for the SNPs g.1884992 T > C (4 exon) of *PAEP*, and g.21643741 G > A (5 exon) of *KRT*10, by using the online tool RNAfold (http://rna.tbi.univie.ac.at/cgi-bin/RNAWebSuite/RNAfold.cgi) (Fig 4 and Fig 5). It is worth noticing that there were some differences in mRNA secondary structures before and after the mutations. The minimum free energy of SNPs g.1884992 T > C changed from −1276.28 kJ/mol to −1287.99 kJ/mol, indicating a decrease. In SNPs g.21643741 G > A, the minimum free energy was −2923.60 kJ/mol before mutation and −2921.69 kJ/mol after mutation, suggesting an increase.

### Predictions of *PAEP* protein secondary and tertiary structures

**Fig 6:**
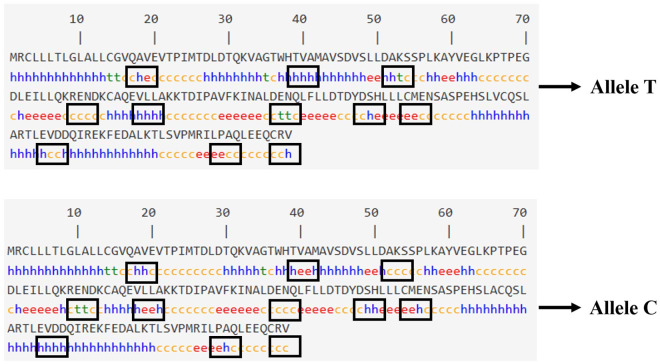
The change of secondary protein structure about SNPs g.1884992 T>C in *PAEP* gene. Note: h in the figure is α-helix, c is a random coil, e is an extended chain, and t is a beta-sheet.

**Fig 7:**
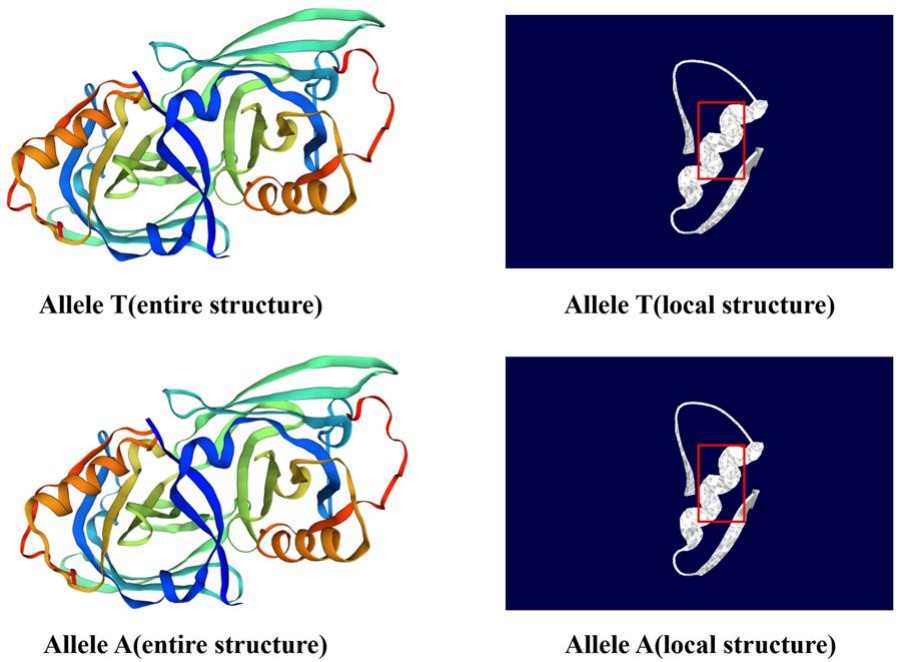
The change of entire and local tertiary protein structure about SNPs g.1884992 T>C in *PAEP* gene.

The secondary structure of the protein encoded by the *PAEP* gene in Kele pigs is shown in Fig 6. Before the SNPs g.1884992 T > C mutation, the percentages of α-helix, beta strand, and random coil were 12.47%, 12.98%, and 67.22%, respectively. After mutation, the percentages of α-helix, beta strand, and random coil were 12.78%, 13.12%, and 66.78%, respectively. Specifically, after the mutation, 11 changes in the protein’s secondary structure were observed, including e (extended chain) transforming into h (α-helix), h (α-helix) changing into e (extended chain), and t (beta-sheet) converting into c (random coil), among others. These changes were highlighted in the figure using black boxes. We employed the online software SWISS-MODEL (https://swissmodel.expasy.org/interactive) and Swiss-PdbViewer software to detect the influence on entire and local tertiary structural protein model caused by SNPs g.1884992 T > C in Fig 7. In the entire structure figure, no significant structural differences were observed between the pre- and post-mutation forms. However, in the local structure figure, we observed a slight narrowing of the protein structure within the red box after the mutation.

### Association between the SNPs, diplotypes of *PAEP* gene and reproductive traits in Kele pigs

The association analysis between SNPs of *PAEP* and reproductive traits was presented in Table 9. In the SNP g.1884992 T > C, the individuals of the TT genotype exhibited a significantly higher total number of piglets born and number of piglets born alive compared to those of the TC genotype (*P < *0.05). In the SNPs g.1885125 G > C and g.1885158 G > A, CC and AA genotypes displayed a significantly higher total number of piglets born, number of piglets born alive, number of piglets weaned, and litter weight weaned than the remaining genotypes (*P < *0.05). GC genotype of SNPs g.1885125 G > C and GA genotype of SNPs g.1885158 G > A had significantly higher numbers of piglets weaned and litter weight weaned compared to GG genotypes (*P < *0.05).

**Table 9:** Association analysis between *PAEP* SNPs and reproductive traits in Kele pigs.

SNPs	Genotype	Total number of piglets born	Number of piglets born alive	Litter birth weight/kg	Number of piglets weaned	Litter weight weaned/kg
**g.1884992** **T > C**	TT (173)	9.38 ± 2.98^a^	8.65 ± 3.03^a^	8.39 ± 3.26	6.98 ± 2.71	37.32 ± 15.40
	TC (65)	7.95 ± 2.16^b^	7.74 ± 2.62^b^	8.63 ± 3.02	6.53 ± 2.28	35.68 ± 13.17
	CC (17)	8.50 ± 1.73^ab^	7.50 ± 1.57^ab^	7.66 ± 0.80	6.00 ± 1.28	40.51 ± 10.33
**g.1885125** **G > C**	GG (153)	8.50 ± 2.46^b^	8.02 ± 2.58^b^	8.26 ± 3.06	6.29 ± 2.18^c^	34.27 ± 12.76^c^
	GC (65)	9.26 ± 2.93^b^	8.42 ± 3.12^b^	8.60 ± 3.24	7.37 ± 2.68^b^	40.64 ± 15.79^b^
	CC (37)	13.50 ± 2.74^a^	13.00 ± 2.19^a^	10.03 ± 1.73	11.50 ± 1.64^a^	60.58 ± 2.71^a^
**g.1885158** **G > A**	GG (153)	8.50 ± 2.46^b^	8.02 ± 2.58^b^	8.26 ± 3.06	6.29 ± 2.18^c^	34.27 ± 12.76^c^
	GA (65)	9.26 ± 2.93^b^	8.42 ± 3.12^b^	8.60 ± 3.24	7.37 ± 2.68^b^	40.64 ± 15.79^b^
	AA (37)	13.50 ± 2.74^a^	13.00 ± 2.19^a^	10.03 ± 1.73	11.50 ± 1.64^a^	60.58 ± 2.71^a^

Note: different shoulder letters between two pairs in the same column mean a significant difference(*P < 0.05*), while others mean no significant difference (*P > 0.05*). The next table is similar.

The correlations between diplotypes of *PAEP* and reproductive traits in Kele pigs were shown in Table 10. For total number of piglets born, individuals with the H3H3 diplotypes revealed a significantly higher value than those of H1H1, H1H2, H1H3, H2H2, H2H3 (*P* < 0.05), and H1H3 diplotypes were significantly higher than H1H1, H1H2, H2H2, H2H3 (*P* < 0.05). There was no significant difference among individuals with H1H1, H1H2, H2H2, and H2H3 diplotypes in the total number of piglets born (*P > *0.05). In number of piglets born alive, H3H3 diplotypes were significantly higher than H1H1, H1H2, H1H3, H2H2, H2H3 (*P < *0.05), and H1H3 diplotypes were significantly higher than H2H3 (*P < *0.05). For litter birth weight, the H1H2 diplotype was significantly higher than H1H1 (*P < *0.05). In the trait of number of piglets weaned, H3H3 diplotypes were significantly higher than H1H1, H1H2, H1H3, H2H2, H2H3 (*P < *0.05), and H1H3 diplotypes were significantly higher than H1H1, H1H2, H2H2, H2H3 (*P < *0.05). For litter weight weaned, H3H3 diplotypes were significantly higher than H1H1, H1H2, H1H3, H2H2, H2H3 (*P < *0.05), and H1H3 diplotypes were significantly higher than H1H1, H1H2, H2H3 (*P < *0.05).

**Table 10:** Correlation between *PAEP* diplotypes and reproductive traits in Kele pigs.

diplotypes	Total number of piglets born	Number of piglets born alive	Litter birth weight/kg	Number of piglets weaned	Litter weight weaned/kg
**H1H1(94)**	8.66 ± 2.62^c^	8.12 ± 2.75^bc^	7.98 ± 3.25^b^	6.13 ± 2.26^c^	32.09 ± 12.49^d^
**H1H2(42)**	8.07 ± 2.25^c^	7.97 ± 2.44^bc^	9.25 ± 2.89^a^	6.81 ± 2.23^c^	37.70 ± 13.12^c^
**H1H3(42)**	10.48 ± 3.01^b^	9.23 ± 3.16^b^	9.20 ± 3.31^ab^	8.36 ± 2.59^b^	46.84 ± 15.36^b^
**H2H2(37)**	8.50 ± 1.73^c^	7.50 ± 1.57^bc^	7.66 ± 0.80^ab^	6.00 ± 1.28^c^	40.51 ± 10.33^bc^
**H2H3(23)**	7.81 ± 2.08^c^	7.46 ± 2.83^c^	7.89 ± 3.06^ab^	6.19 ± 2.33^c^	33.26 ± 13.07^cd^
**H3H3(17)**	13.50 ± 2.74^a^	13.00 ± 2.19^a^	10.03 ± 1.73^ab^	11.50 ± 1.64^a^	60.58 ± 2.71^a^

### Association between the SNPs, diplotypes of *KRT*10 gene and reproductive traits in Kele pigs

According to the result in Table 11, the CC genotype of the SNPs g.21643703 C > T had significantly higher values in litter birth weight and litter weight weaned compared to those of CT and TT genotypes (*P* < 0.05), which also had significantly higher number of piglets weaned compared to CT genotype (*P* < 0.05). In the SNPs g.21643714 G > A, the AA genotype exhibited a significantly higher total number of piglets born and litter weight weaned than the GA genotypes (*P* < 0.05). However, there were no significant differences in the number of piglets born live, litter birth weight, and number of piglets weaned in SNPs g.21643714 G > A (*P* > 0.05). In the SNPs g.21643741 G > A, the GG genotype showed a significantly higher total number of piglets born, number of piglets born alive, number of piglets weaned, and litter weight weaned compared to AA genotypes (*P* < 0.05). Meanwhile, it had a significantly higher total number of piglets born compared to GA genotypes (*P* < 0.05).

**Table 11:** Association analysis between *KRT*10 SNPs and reproductive traits in Kele pigs.

SNPs	Genotypes	Total number of piglets born	Number of piglets born alive	Litter birth weight/kg	Number of piglets weaned	Litter weight weaned/kg
**g.21643703** **C > T**	CC (115)	8.83 ± 2.74	8.49 ± 2.51	8.88 ± 2.69^a^	7.26 ± 2.29^a^	41.33 ± 14.22^a^
	CT (113)	8.75 ± 2.57	8.27 ± 2.82	8.07 ± 3.42^b^	6.36 ± 2.52^b^	35.29 ± 15.35^b^
	TT (27)	8.56 ± 2.81	7.44 ± 3.12	7.03 ± 2.9^b^	6.44 ± 3.23^ab^	32.83 ± 13.17^b^
**g.21643714** **G > A**	GG (200)	7.90 ± 2.18^ab^	7.60 ± 1.71	7.81 ± 0.84	6.40 ± 1.27	35.57 ± 1.95^ab^
	GA (45)	7.87 ± 3.02^b^	7.67 ± 3.13	8.01 ± 3.87	6.27 ± 2.10	33.81 ± 13.91^b^
	AA (10)	9.01 ± 2.55^a^	8.46 ± 2.66	8.43 ± 2.98	6.91 ± 2.66	38.74 ± 15.41^a^
**g.21643741** **G > A**	GG (162)	10.4 ± 1.24^a^	9.80 ± 1.01^a^	9.58 ± 2.43	8.20 ± 1.37^a^	47.42 ± 6.03^a^
	GA (78)	8.87 ± 2.83^b^	8.41 ± 2.68^ab^	8.39 ± 2.53	7.04 ± 2.54^ab^	39.86 ± 14.73^a^
	AA (15)	8.56 ± 2.63^b^	8.08 ± 2.82^b^	8.18 ± 3.38	6.52 ± 2.57^b^	35.84 ± 15.20^b^

As shown in Table 12, for total number of piglets born, the value of H3H3 diplotype was significantly higher than those of H1H4, H2H2, H2H3, H3H4, H4H4 (*P < *0.05), and H1H1, H1H2, H1H3, H2H4 diplotypes were significantly higher than H1H4, H3H4, H4H4 (*P < *0.05). Regarding number of piglets born alive, H3H3 diplotype was significantly higher than H1H4, H2H2, H3H4, H4H4 (*P < *0.05), and H1H1, H1H2, H1H3, H2H3, H2H4 diplotypes were significantly higher than H1H4, H3H4 (*P < *0.05). For litter birth weight, H1H1 diplotype was significantly higher than H1H2, H1H4, H2H2, H3H4, H4H4 (*P < *0.05), and H1H3, H2H3, H2H4, H3H3, H4H4 diplotypes were significantly higher than H2H2, H3H4 (*P < *0.05). In the trait of the number of piglets weaned, the H3H3 diplotype was significantly higher than H1H2, H1H4, and H3H4 (*P < *0.05). For litter weight weaned, H1H1and H3H3 diplotypes were significantly higher than H1H2, H1H4, H2H2, H2H4, H4H4 (*P < *0.05).

**Table 12:** Correlation between *KRT*10 diplotypes and reproductive traits in Kele pigs.

diplotypes	Total number of piglets born	Number of piglets born alive	Litter birth weight/kg	Number of piglets weaned	Litter weight weaned/kg
**H1H1 (29)**	9.00 ± 2.28^ab^	8.90 ± 2.57^ab^	10.33 ± 3.47^a^	7.72 ± 2.07^ab^	46.58 ± 15.2^a^
**H1H2 (60)**	8.65 ± 2.50^ab^	8.10 ± 2.63^ab^	7.64 ± 3.01^bc^	6.15 ± 2.75^b^	33.87 ± 15.79^b^
**H1H3 (35)**	9.97 ± 3.05^ab^	9.37 ± 2.56^ab^	8.83 ± 1.52^ab^	7.60 ± 2.89^ab^	41.30 ± 14.39^ab^
**H1H4 (17)**	6.65 ± 1.90^d^	6.47 ± 1.91^c^	7.67 ± 2.23^bc^	5.94 ± 1.71^b^	32.23 ± 17.33^b^
**H2H2 (27)**	8.56 ± 2.81^b^	7.44 ± 3.12^bc^	7.03 ± 2.90^c^	6.44 ± 3.23^ab^	32.83 ± 13.17^b^
**H2H3 (34)**	8.41 ± 2.16^bc^	7.97 ± 2.56^ab^	8.37 ± 2.98^ab^	6.65 ± 2.15^ab^	38.90 ± 16.08^ab^
**H2H4 (19)**	9.68 ± 3.30^ab^	9.37 ± 3.64^ab^	8.92 ± 5.03^ab^	6.53 ± 2.44^ab^	33.28 ± 11.81^b^
**H3H3 (15)**	10.40 ± 1.24^a^	9.80 ± 1.01^a^	9.58 ± 2.43^ab^	8.20 ± 1.37^a^	47.42 ± 6.03^a^
**H3H4 (9)**	6.33 ± 2.18^d^	6.33 ± 2.18^c^	6.72 ± 3.38^c^	6.33 ± 2.18^b^	37.92 ± 11.15^ab^
**H4H4 (10)**	7.90 ± 2.18^cd^	7.60 ± 1.71^bc^	7.81 ± 0.84^b^	6.40 ± 1.27^ab^	35.57 ± 1.95^b^

### Association between the SNPs, diplotypes of *BMP*7 gene and reproductive traits in Kele pigs

For the *BMP*7 gene (Table 13), individuals with the AG genotype of SNPs g.57647887 G > A had a significantly higher total number of piglets born and number of piglets born live compared to AA genotypes (*P* < 0.05). In SNPs g.57647990 C > T, the CT genotype exhibited a significantly higher total number of piglets born and number of piglets born live than the TT genotypes (*P* < 0.05). GG genotype displayed significantly higher litter weight weaned compared to CC genotypes in SNPs g.57648145 C > G (*P* < 0.05).

**Table 13:** Association analysis between *BMP*7 SNPs and reproductive traits in Kele pigs.

SNPs	Genotypes	Total number of piglets born	Number of piglets born alive	Litter birth weight/kg	Number of piglets weaned	Litter weight weaned/kg
**g.57647887** **G > A**	GG (95)	8.00 ± 2.60^ab^	7.26 ± 2.75^ab^	7.68 ± 2.45	6.26 ± 2.02	36.49 ± 13.74
	GA (102)	9.09 ± 2.12^a^	8.26 ± 2.22^a^	8.07 ± 3.15	6.86 ± 2.76	36.68 ± 13.21
	AA (58)	8.15 ± 3.34^b^	7.77 ± 3.45^b^	7.65 ± 3.27	6.15 ± 2.71	34.56 ± 16.99
**g.57647990** **C > T**	CC (95)	8.00 ± 2.60^ab^	7.26 ± 2.75^ab^	7.68 ± 2.45	6.26 ± 2.02	36.49 ± 13.74
	CT (102)	9.09 ± 2.12^a^	8.26 ± 2.22^a^	8.07 ± 3.15	6.86 ± 2.76	36.68 ± 13.21
	TT (58)	8.15 ± 3.34^b^	7.77 ± 3.45^b^	7.65 ± 3.27	6.15 ± 2.71	34.56 ± 16.99
**g.57648145** **C > G**	CC (79)	8.71 ± 2.87	8.33 ± 2.93	7.72 ± 2.76	6.13 ± 2.21	34.31 ± 13.05^b^
	CG (106)	8.5 ± 2.22	7.36 ± 2.56	7.81 ± 3.00	6.50 ± 2.62	35.25 ± 14.31^ab^
	GG (70)	8.26 ± 2.86	7.9 ± 2.52	8.09 ± 2.95	7.00 ± 2.55	40.61 ± 14.08^a^

The association analysis of *PAEP* gene diplotypes with reproductive traits in Kele pigs was shown in Table 14. For total number of piglets born, H1H1, H1H2, H1H3, H1H4, H2H2, H2H3, H3H3, H4H4 diplotypes were significantly higher than H2H4 (*P < *0.05). In the trait of number of piglets born alive, H1H1, H1H2, H1H4, H2H2, H2H3, H3H3, H4H4 diplotypes were significantly higher than H2H4 (*P < *0.05). For litter birth weight, H4H4 diplotypes were significantly higher than H1H4, H2H4, H3H3 (*P < *0.05). In the trait of number of piglets weaned, H1H1, H1H2, H1H3, H1H4, H2H2, H2H3, H3H3, H4H4 diplotypes were significantly higher than H2H4 (*P < *0.05). For litter weight weaned, H1H1, H1H2, H1H3, H1H4, H2H2, H2H3, H3H3, H4H4 diplotypes were significantly higher than H2H4 (*P < *0.05).

**Table 14:** Correlation between *BMP*7 diplotypes and reproductive performance in Kele pigs.

diplotypes	Total number of piglets born	Number of piglets born alive	Litter birth weight/kg	Number of piglets weaned	Litter weight weaned/kg
**H1H1 (39)**	8.36 ± 2.92^a^	7.93 ± 2.54^a^	8.04 ± 2.44^ab^	7.21 ± 1.85^a^	42.49 ± 11.89^a^
**H1H2 (57)**	9.22 ± 1.89^a^	8.17 ± 2.16^a^	8.13 ± 2.92^ab^	7.17 ± 2.58^a^	37.67 ± 12.37^a^
**H1H3 (36)**	7.50 ± 2.11^a^	6.08 ± 2.65^ab^	7.72 ± 2.77^ab^	5.67 ± 1.97^a^	33.18 ± 15.32^a^
**H1H4 (16)**	7.33 ± 3.14^a^	7.00 ± 2.68^a^	6.75 ± 4.27^b^	6.00 ± 4.98^a^	31.38 ± 23.10^a^
**H2H2 (31)**	8.40 ± 3.44^a^	7.90 ± 3.60^a^	7.69 ± 2.59^ab^	6.50 ± 2.40^a^	36.22 ± 16.11^a^
**H2H3 (29)**	9.33 ± 2.17^a^	8.89 ± 2.14^a^	8.37 ± 3.40^ab^	6.33 ± 2.28^a^	35.92 ± 11.53^a^
**H2H4 (13)**	4.00 ± 1.77^b^	4.00 ± 2.42^b^	1.70 ± 1.28^c^	1.00 ± 1.56^b^	4.50 ± 8.00^b^
**H3H3 (20)**	8.20 ± 2.78^a^	8.20 ± 2.78^a^	6.61 ± 1.21^b^	5.00 ± 1.33^a^	27.60 ± 5.19^a^
**H4H4 (14)**	9.00 ± 2.31^a^	9.00 ± 2.31^a^	10.43 ± 3.55^a^	7.00 ± 2.31^a^	41.33 ± 8.86^a^

## Discussion

*PAEP* is a protein initially described in reproduction. Furthermore, *PAEP* existed in three isoforms, each contributing to distinct molecular pathways that regulated the uterine environment for pregnancy maintenance, initiation, and prolongation by modulating endometrial receptivity and immune tolerance, making it a potential biomarker for assisted reproductive technologies [41–44]. In this study, we detected the SNPs g.1884992 T > C (exon 4), g.1885125 G > C (intron 4), and g.1885158 G > A (intron) in *PAEP*. The moderate effective number of alleles suggested balanced allele distribution, enabling populations to maintain adaptability [45]. The SNPS g.1885125 G > C and g.1885158 G > A with moderate heterozygosity helped maintain adaptability, prevented inbreeding depression caused by high homozygosity, and preserved a certain level of genetic diversity to cope with environmental selection pressure [46]. The SNP g.1884992 T > C with high heterozygosity contributed to enriching the gene pool of the population [39,45]. The SNPs g.1884992 T > C, g.1885125 G > C, and g.1885158 G > A all highly significantly deviated from Hardy-Weinberg equilibrium (*P* < 0.01), suggesting that gene distribution may be influenced by natural selection, drift, migration, and other factors [46]. The SNP of g.1884992 T > C was in the fourth exon located in the gene’s coding sequence region, which may alter the protein’s conformation. The prediction result of the mRNA secondary structure showed that the free energy of the mRNA secondary structure was reduced, and the structural stability was enhanced after the mutation. The mRNA synthesizes proteins using itself as a template, and changes in mRNA may have an impact on the structure and function of proteins. We made further predictions of the secondary and tertiary structures of the PAEP protein to test that. The result showed that the secondary structure of the protein encoded by the SNPs g1884992 T > C changed, and the tertiary structure was similar. The side chains of valine (V) and alanine (A) are both hydrophobic and have similar sizes, which may be the reason why their protein tertiary structures are similar [47,48]. However, there are still significant differences in their biological functions and chemical properties, and their functional expression may change in the reproductive traits of Kele pigs. The significant differences in total litter size, live litter size, weaned piglets, and weaned litter weight after the mutation may be the result of changes in protein structure and function. The SNPs g.1885125 G > C and g.1885158 G > A were both located in the fourth intron. While mutations in this region did not alter the coding sequence, previous studies have suggested that intronic variants can influence gene expression by affecting transcriptional regulation or alternative splicing [49–52]. However, further experimental validation is required to confirm whether these SNPs have functional effects on gene expression and transcriptional activity. The results of the correlation analysis showed that the two SNPs had an impact on reproductive traits, such as total litter size, live litter size, and weaned piglet size in Kele pigs. In this study, the individuals with the CC genotype of SNPs g. 1885152 G > C and AA genotype of SNPs g.1885158 G > A had a significantly higher total number of piglets born, number of piglets born alive, number of piglets weaned, and litter weight weaned than other genotypes, indicating a favorable genotype. This is consistent with the experimental results of Tsiaras et al. [16], whose study indicated that Holstein cows with the AA genotype in PAEP had a significantly shorter gestation length than those with the AB or BB genotype, making it a favorable genotype for improving reproductive traits in cattle. While the specific molecular pathways require further investigation, these results collectively highlight *PAEP* as a candidate gene for improving reproductive performance in livestock. The analysis of diplotype H3H3 (CCGGGG) individuals showed that total number of piglets born, number of piglets born alive, number of piglets weaned, and litter weight weaned were significantly higher than H1H1 (TTGGGG), H1H2 (TTGCGA), H1H3 (CTGGGG), H2H2 (TTCCAA), and H2H3 (TCGCGA) (*P* < 0.05), indicating a favorable diplotype. The diplotype H3H3 (CCGGGG) can serve as a reference for molecular marker-assisted selection in the total number of piglets born, number of piglets born alive, number of piglets weaned, and litter weight weaned.

As genes encoding important proteins in keratinocytes, *KRT*10 and its homologs have not been reported to exhibit polymorphism in reproduction traits of livestock and poultry. However, their significant expression in reproductive organs [53–55] suggests potential involvement in key physiological processes. The keratin family contributes to epithelial integrity, cellular differentiation, and immune regulation, which are fundamental mechanisms for reproductive organ function [56,57]. Notably, keratins interact with signaling pathways such as TGF-β and Wnt/β-catenin [58–60], which regulate epithelial homeostasis, endometrial receptivity, and embryo implantation by modulating cellular proliferation, adhesion, and immune tolerance. Based on these molecular interactions, variations in keratin gene polymorphisms may influence pathway activity, potentially impacting reproductive traits such as fertility, implantation success, and litter size. There were three SNPs in *KRT*10*,* including g.21643703 C > T (intron 4), g.21643714 G > A (intron 4), and g.21643741 G > A (exon 5)*.* The SNP g.21643714 G > A exhibited low genetic diversity and heterozygosity, indicating that individuals in this population rarely appear as heterozygotes at these SNP sites [61]. The SNPs g.21643714 G > A showed low polymorphism (*PIC* < 0.25). It indicated the variant alleles or genotypes of the *KRT*10 gene in the Koala pig population are relatively stable, with fewer variations, potentially resulting in lower adaptability to environmental changes [62]. The SNPs g.21643703 C > T and g.21643741 G > A exhibited moderate polymorphism, indicating a reasonable level of genetic variation within the population. This suggested that these SNPs may be subject to balancing selection, where natural selection maintains the coexistence of two or more alleles rather than eliminating a specific allele [63]. Chi-square tests showed that the genotyping in SNPs g.21643703 C > T and g.21643741 G > A were in Hardy-Weinberg equilibrium (*P* > 0.05), indicating the absence of evolutionary influences such as natural selection, mutation, migration, and genetic drift [64]. The SNP g.21643714 G > A exhibited a highly significant deviation from Hardy-Weinberg equilibrium. This may be due to the fact that the experimental population consists of a local pig breed, where factors such as genetic drift, migration and historical selection pressures have contributed to random fluctuations in allele frequencies across generations, leading to a reduction in heterozygosity and a significant deviation from Hardy-Weinberg equilibrium [65]. The three SNPs did not exhibit strong linkage disequilibrium, indicating that the alleles at these SNPs were independently distributed within the population without a significant pattern of co-inheritance [66]. We analyzed the molecular biological structure encoded by the SNPs g. 21643741 G > A in the fifth exon. Before and after the mutation, the amino acids encoded by SNPs g.21643741 G > A both were lysine (K), indicating a synonymous mutation. Synonymic mutation refers to the phenomenon of base replacement without causing changes in amino acid types. Previous studies have demonstrated that synonymous mutations can affect gene expression by altering mRNA stability, secondary structure, splicing, and translation dynamics [67–69]. In our study, the prediction result of the mRNA secondary structure found that the structure changed and the structural stability weakened after mutation. This finding suggested that the SNP g.21643741 G > A may have potential functional implications and warrant further investigation. In this study, the significant differences in total number of piglets born, number of piglets born alive, number of piglets weaned, and litter weight weaned of the SNPs g. 21643741 G > A may be the result of synonymous mutations. Individuals with the GG genotype in SNPs 21643741 G > A had significantly higher total number of piglets born, number of piglets born alive, number of piglets weaned, and litter weight weaned than those with the AA genotype (*P* < 0.05), and significantly higher total number of piglets born than those with the GA genotype (*P* < 0.05). It was preliminarily identified as a favorable genotype. Molecular breeding techniques can be latterly used to enhance the selection intensity of this locus and achieve homozygous genotype GG as soon as possible to improve reproductive traits. The analysis of the diplotypes showed that individuals with H1H1 (CCGGGGG) had higher litter birth weight, and it was the optimal diplotype for increasing litter birth weight. The individuals of the H3H3 (CCGGAA) diplotype reached significant levels in all traits, indicating a favorable diplotype and serving as a reference for molecular marker-assisted selection.

Existing research indicates that the *BMP*7 gene has significant implications for improving reproductive traits in livestock and poultry. Xu et al. [70] found that the mRNA expression level of *BMP*7 in the follicles of multi-lamb Hu sheep was significantly higher than that of single-lamb individuals. Zhang et al. [71] found that changes involving transcription factors such as USF1, USF2, and INMS1 in the *BMP*7 promoter region might be involved in greater sheep prolificacy. In the study by Yin et al. [37], the SNPs c.1569 A > G in *BMP*7 had a significant impact on some reproductive traits of sows in large white pig populations, which had some similarities with the results of this study. We found the SNPs g.57647887 G > A, g.57647990 C > T, and g.57648145 C > G in *BMP*7, all in intron 3. All SNPs exhibited low heterozygosity, intermediate polymorphism (*PIC* < 0.25), and highly significantly deviated from Hardy-Weinberg equilibrium (*P* < 0.01). The SNPs g.57647887 G > A and g.57647990 C > T exhibited significant levels on the total number of piglets born and number of piglets born alive (*P* < 0.05), and the individuals with GA genotype and CT genotype had higher values than others. The SNP g.57648145 C > G significantly influenced litter weight weaned (*P* < 0.05), and the individuals with the GG genotype had higher values than others. Individuals with the H4H4 diplotypes exhibited significant effects on the total number of piglets born, number of piglets born alive, litter birth weight, number of piglets weaned, and litter weight weaned, indicating it can be a favorable diplotypes.

## Conclusion

In this study, we totally identified nine SNPs in *PAEP*, *KRT*10, and *BMP*7 genes. Through a comprehensive analysis, it was observed that the H3H3 (CCGGGG) diplotype of *PAEP* and the H3H3 (CCGGAA) diplotype of *KRT*10 could be used as a potential genetic marker to improve the total number of piglets born, number of piglets born alive, number of piglets weaned, and litter weight weaned. Additionally, the H4H4 (AATTGG) diplotype of *BMP*7 could be used as a molecular marker for assisting in the selection of the total number of piglets born, number of piglets born alive, litter birth weight, number of piglets weaned, and litter weight weaned. Consequently, the *PAEP*, *KRT*10, and *BMP*7 could all be candidate genes for the reproductive traits of Kele pigs.

## Supporting information

S1 Appendix *PAEP* sequencing results.
(ZIP)

S2 Appendix *KRT10* sequencing results.
(ZIP)

S3 Appendix *BMP7* sequencing results.
(ZIP)

S4 Appendix Minimal date set.
(XLS)
